# Age, source, and future risk of COVID-19 infections in two settings of Hong Kong and Singapore

**DOI:** 10.1186/s13104-020-05178-z

**Published:** 2020-07-13

**Authors:** Shuying Zhu, Jun Tao, Huizhi Gao, Daihai He

**Affiliations:** 1 School of Public Health, LKS Faculty of Medicine, The University of Hong Kong, Hong Kong, Hong Kong SAR China; 2 Department of Applied Mathematics, Hong Kong Polytechnic University, Hong Kong, Hong Kong SAR China

**Keywords:** COVID-19, Age structure, Hong kong, Singapore

## Abstract

**Objective:**

To explore and compare the age, source and future risk of COVID-19 infection in Hong Kong SAR China and Singapore as of March 5, 2020.

**Results:**

We find significant difference in age patterns of confirmed cases in these 2 localities early in the pandemic.

**Conclusion:**

We highlight the potential importance of population age structure in confirmed cases, which should be considered in evaluation of the effectiveness of control effort in different localities.

## Introduction

The coronavirus disease 2019 (COVID-19) pandemic first broke out in Wuhan, China in December 2019, and was declared as a global pandemic by the World Health Organization (WHO) on 11 March 2020 [1]. As of 15 June 2020, more than 7,000,000 confirmed cases and approximately 5.5% mortality were identified worldwide [2]. The control experience in China showed that the isolation of infected persons, the tracking and quarantine of susceptible individuals, and other social distancing measures could contain this epidemic [3]. Singapore and Hong Kong are both praised as having taken “very effective” measures to intervene in the transmission of new coronavirus by a WHO official [4]. As of March 5, there were 112 cases in Singapore and 106 cases in Hong Kong. It seemed that there was no significant difference in these 2 numbers given the fact that the measures adopted by Singapore were not as strict as those adopted by Hong Kong but both cities were at high importation risk due to the high proportion of mainland Chinese population flow. Therefore, some media held the view that Singapore adopted a plausibly proper but very effective strategy. In order to scientifically investigate the difference in the effectiveness of Singapore and Hong Kong, we performed several statistical tests of the new coronavirus cases of Singapore and Hong Kong in regard to their age distributions as of March 5. We found that the situation in Singapore at this early point in the pandemic may have attributed to the relatively large proportion of infection in the younger generations of its population. However, no significant differences between the effectiveness of the measures adopted by Hong Kong and Singapore government at this time period were noted.

## Methods

We obtained the data of the COVID-19 cases of Singapore and Hong Kong from Singapore and Hong Kong’s governmental websites [5, 6]. The imported cases and local cases of both districts were studied separately. And we obtained the age structure data of Hong Kong and Singapore separately from public websites as of March 5. In order to compare the difference of age distributions of the 2 infected population and the effects of age structure. We performed the Wilcoxon rank-sum test to compare the age distributions of the infected patients in Hong Kong with those in Singapore. And then we divided age into age groups, and we counted the number of cases in each age group. We then used the Wilcoxon rank-sum test to compare the difference of age-specific incidence between the 2 populations. Lastly, we obtained the age-specific incidence rate by dividing the number of cases in each age group by the population of that group and then compared the age-specific incidence rate by the Chi-square test.

## Results and discussion

**Fig. 1:**
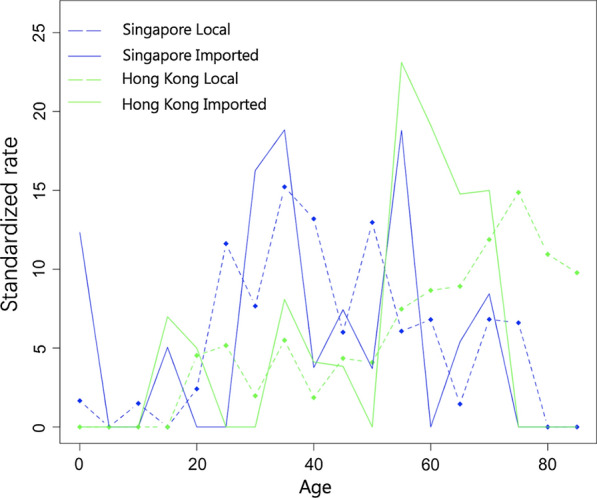
Standardized incidence rate of the 4 groups

From the onset of COVID-19 to March 5, there have been 23 imported cases in Singapore and Hong Kong, respectively. The age distribution of imported and local cases can be seen in Additional file 1: Table S1 and Figure S1, which was similar to an epidemiological analysis using outsourced data in Mainland China [7]. Considering that Hong Kong has a total population of 7.45 million and is more geographically close to Mainland China, the risk of importing COVID-19 cases should be higher than Singapore which has a total population of 5.64 million and less pressure of population mobility with Mainland China. Results by Wilcox test reported a significant difference in age patterns of both imported cases and local cases between the 2 places, with Hong Kong taking up a higher percentage of elderly cases. Nevertheless, this difference is no longer significant after the standardization of imported and local case numbers by each age category in Hong Kong and Singapore respectively, shown in Table 1 and Fig. 1. Therefore, it is reasonable to assume that the variation in age distribution of the cases might arise from the age structure of the population per se. Appropriate travel restrictions may delay the introduction of COVID-19 infection. Health authorities can provide advice or warnings to travelers. The formulation of the policy must weigh the potential serious economic consequences and the effect of the pandemic control.

**Table 1:** The results of statistical tests as of March 5

	HK vs Singapore local cases	HK vs Singapore imported cases	HK local vs imported cases	Singapore local vs imported cases
*p* value of age distribution	0.00139	0.000398	0.624	0.874
*p* value of age-specific cases	0.000954	0.839	0.00471	0.0186
*p* value of age-specific incidence rate	0.111	0.00573	0.0973	0.0746

## Conclusion

Since the elderly are more vulnerable to get infected after exposure and develop into severe cases, thus the population age structure, among other factors, should be considered when comparing the effectiveness of control measure in different localities.

## Limitations

The situations of COVID-19 change fast worldwide. The COVID-19 data of Singapore with age information has been unavailable for public after we first submitted this work. Hence, our work performed on merely the data of Singapore and Hong Kong as of March 5. Although the patterns of infected cases may stay similar to some extent, the age structures of infected cases in both locations may be different now.

## Supplementary information

**Additional file 1: Table S1** The cases of Hong Kong and Singapore by March 5. **Figure S1** Histogram of local/imported case numbers by age distribution in HK and Singapore by March 5.

## Data Availability

All data and materials used in this work were publicly available.

## Abbreviations

COVID-19: Coronavirus disease 2019
WHO: World Health Organization
