# Supplementary material for: Age, source, and future risk of COVID-19 infections in two settings of Hong Kong and Singapore
Source: BMC Res Notes. 2020 Jul 13;13:336. doi: 10.1186/s13104-020-05178-z (PMC7356138; doi:10.1186/s13104-020-05178-z)
Supplement: Supplementary file 1 — Additional file 1: Table S1 The cases of Hong Kong and Singapore by March 5. Figure S1 Histogram of local/imported case numbers by age distribution in HK and Singapore by March 5. [file 13104_2020_5178_MOESM1_ESM.docx]

Supplementary Material: Table and Figure of raw data

Table S1: The cases of Hong Kong and Singapore by March 5

|  | Singapore | | | | Hong Kong | | | |
| --- | --- | --- | --- | --- | --- | --- | --- | --- |
|  | Local | | Imported | | Local | | Imported | |
| Age | cases | standardized incidence rate | cases | standardized incidence rate | cases | standardized incidence rate | cases | standardized incidence rate |
| 0-4 | 1 | 12.3 | 2 | 1.66 | 0 | 0.00 | 0 | 0.00 |
| 5-9 | 0 | 0 | 0 | 0.00 | 0 | 0.00 | 0 | 0.00 |
| 10-14 | 1 | 0 | 0 | 1.49 | 0 | 0.00 | 0 | 0.00 |
| 15-19 | 0 | 5.05 | 1 | 0.00 | 0 | 0.00 | 1 | 6.99 |
| 20-24 | 2 | 0 | 0 | 2.41 | 4 | 4.53 | 1 | 5.00 |
| 25-29 | 11 | 0 | 0 | 11.62 | 5 | 5.17 | 0 | 0.00 |
| 30-34 | 7 | 16.3 | 4 | 7.67 | 2 | 1.98 | 0 | 0.00 |
| 35-39 | 15 | 18.8 | 5 | 15.22 | 6 | 5.49 | 2 | 8.09 |
| 40-44 | 13 | 3.77 | 1 | 13.19 | 2 | 1.86 | 1 | 4.12 |
| 45-49 | 6 | 7.43 | 2 | 6.01 | 5 | 4.35 | 1 | 3.84 |
| 50-54 | 13 | 3.70 | 1 | 12.97 | 5 | 4.09 | 0 | 0.00 |
| 55-59 | 6 | 18.8 | 5 | 6.07 | 10 | 7.47 | 7 | 23.11 |
| 60-64 | 6 | 0 | 0 | 6.81 | 10 | 8.66 | 5 | 19.12 |
| 65-69 | 1 | 5.39 | 1 | 1.45 | 8 | 8.92 | 3 | 14.77 |
| 70-74 | 3 | 8.43 | 1 | 6.82 | 7 | 11.88 | 2 | 14.99 |
| 75-79 | 2 | 0 | 0 | 6.60 | 6 | 14.87 | 0 | 0.00 |
| 80-84 | 0 | 0 | 0 | 0 | 4 | 10.94 | 0 | 0.00 |
| 85+ | 0 | 0 | 0 | 0 | 4 | 9.78 | 0 | 0.00 |


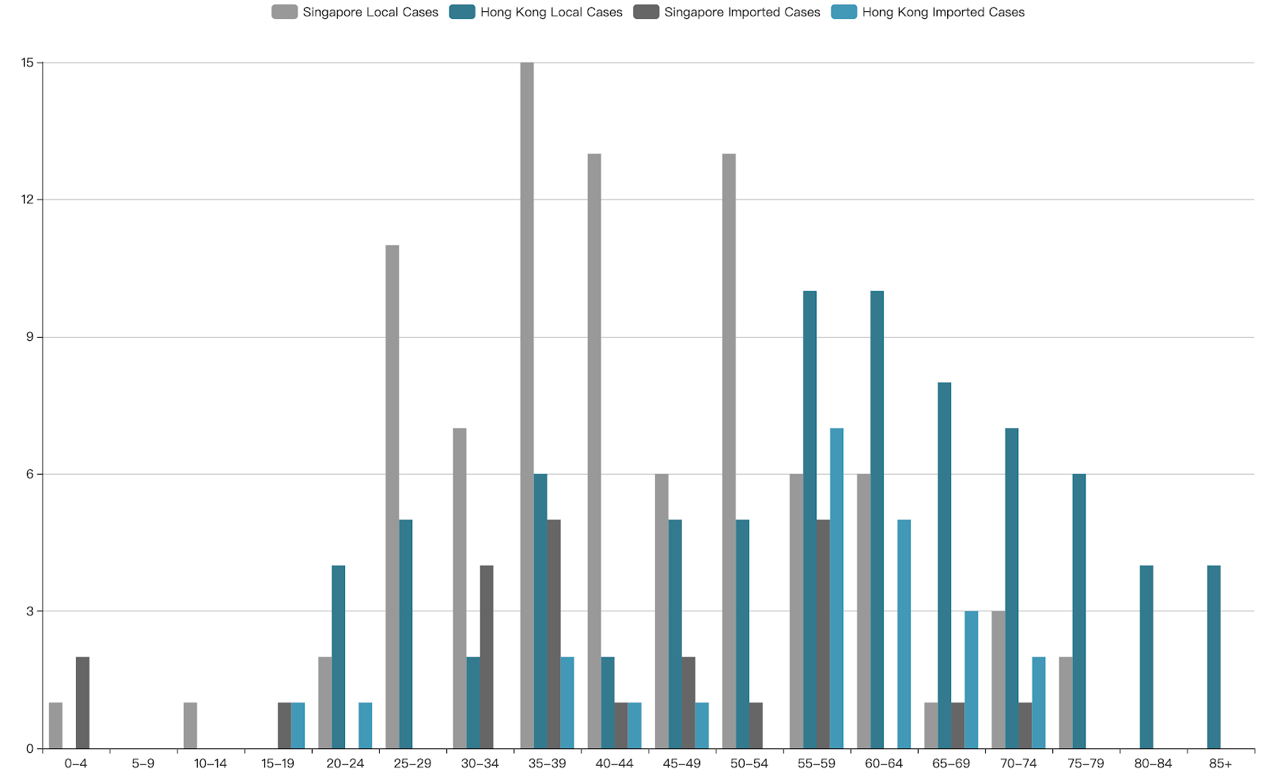


Figure S1. Histogram of local/imported case numbers by age distribution in HK and Singapore by March 5
